# Association Between Work and Common Mental Disorders in School Adolescents: The ERICA Study

**DOI:** 10.6061/clinics/2020/e1794

**Published:** 2020-10-21

**Authors:** Daniele Baptista dos Santos, Mauro Felippe Felix Mediano, Luiz Fernando Rodrigues Júnior, Braulio dos Santos, Andrea Rocha de Lorenzo, Maria Cristina Caetano Kuschnir

**Affiliations:** I Departamento de Pesquisa e Educacao, Instituto Nacional de Cardiologia, Ministerio da Saude, Rio de Janeiro, RJ, BR; II Instituto Nacional de Infectologia Evandro Chagas, Fundacao Oswaldo Cruz, Rio de Janeiro, RJ, BR; III Faculdade de Medicina, Universidade Estacio de Sa, Rio de Janeiro, RJ, BR

**Keywords:** Mental Health, Minor Labor, Adolescent Health, Blood Pressure

## Abstract

**OBJECTIVES:**

Adolescence is characterized by continuing profound mental, physical, and social changes and entering into the labor market during this phase may have negative consequences on physical and mental health. Common mental disorders (CMD) are characterized as disorders of mental functions, including symptoms of depression and anxiety as well as various nonspecific and somatic complaints such as reduced ability to concentrate, tiredness, irritation, and forgetfulness. Despite its increasing prevalence, few studies have addressed CMD and its association with work, in adolescents. In the present study, we aimed to identify the main factors associated with CMD and evaluated its association with work, in school adolescents.

**METHODS:**

A cross-sectional study was conducted with 12 to 17-year-old adolescent students using a self-administered questionnaire with questions related to work. CMD was verified using the General Health Questionnaire of 12 items. In total, 3424 adolescents were studied.

**RESULTS:**

The prevalence of CMD and work in the last year was 28.72% and 19.63%, respectively. After adjustment for potential confounding variables, multivariate analysis showed associations of CMD with female gender (OR=2.72) and work (OR=1.70).

**CONCLUSION:**

In the present study, a high number of cases of CMD were observed among the studied adolescents. Female gender and work history in the last year were negatively and independently associated with the presence of CMD.

## INTRODUCTION

Adolescence is a phase of human development marked by continuing profound changes at the mental, physical, and social levels that requires adolescents to break from their past beliefs and move toward their personal and professional growth by making choices of life independently (1,2). In some countries, adolescents can enter the job market; recent estimates indicate that about 3.4 million children and adolescents, between 10 and 17 years of age, work in Brazil. Brazilian law currently permits adolescents between 16 and 18 years of age to undertake labor activities with their social security rights guaranteed. The law also allows adolescents aged between 14 and 16 years to enter the job market as apprentices in technical and professional training programs. However, although labor by children and adolescents under 14 years old is prohibited by law in Brazil, it is still observed in some circumstances (3).

The entry of adolescents into the job market may be associated with a series of negative implications for their development, increasing their daily stress to a level that adolescents are not always psychologically prepared for and introducing adverse factors into their life by preventing them from performing the ludic and social activities common at their age. Moreover, the involvement of adolescents in the job market can have adverse consequences ranging from poor performance and dropping out of school to abnormalities in physical and mental health (4,5). Other inherent risks for adolescent workers are work-related accidents during transportation to and from the workplace or resulting from a poor work environment (6).

Common mental disorders (CMD), also known as non-psychotic disorders, are characterized by symptoms of depression and anxiety, besides various nonspecific and somatic complaints such as reduced ability to concentrate, tiredness, irritation, and forgetfulness (7). In recent decades, there has been an increase in its prevalence, not only in adults but also among adolescents, that is associated with numerous sociodemographic and environmental factors such as gender, income, education, smoking, and a sedentary lifestyle (8-10).

Currently, there are a growing number of studies in the literature evaluating the relationship between mental health and work. This fact can be explained by the increased prevalence of mental and behavioral disorders in workers in several countries, including Brazil (11-13). However, studies on this topic, in adolescents, are still scarce, which is an important gap in the literature. Thus, in the present study, we aimed to identify the main factors associated with CMD and evaluate its association with work, in school adolescents.

## METHODS

### Study Design

The Study of Cardiovascular Risks in Adolescents (ERICA) was a national multicenter cross-sectional research project conducted with 12 to 17-year-old adolescent students enrolled in morning or afternoon shifts in public and private schools in municipalities with more than 100,000 inhabitants. The primary objective was to estimate the prevalence of cardiovascular risk factors and metabolic syndrome in adolescents (14). In addition, a database with relevant information was compiled during the ERICA study, on the quality of physical and mental health of school adolescents throughout Brazil. The details of the sample size calculation, criteria for selecting schools, and other instruments used, as well as the procedures on the day of data collection, can be found in other publications (14,15).

In the present study, we used information from the ERICA study regarding the presence of CMD and work characteristics of the adolescents from schools located in the municipality of Rio de Janeiro. We included adolescents who did not present physical or mental disabilities that would preclude assessment of the investigated parameters and those who were not pregnant. The data were collected using a self-administered questionnaire comprising 103 questions divided into 11 blocks (sociodemographic aspects, occupational activities, physical activity, eating behavior, smoking, alcohol use, reproductive health, oral health, sleep duration, physical morbidity, and mental health) and were loaded on an electronic portable device. The application of the questionnaire was supervised by a school teacher or members of the study support team. The interviewee could contact any of them in case of questions.

All participating students signed the consent form and brought the free and informed consent form signed by their guardians. The study was approved by the Research Ethics Committee of the coordinating institution and each local center (IESC/UFRJ-Process 45/2008).

### Common Mental Disorder (Outcome)

The CMD evaluation was performed using the Brazilian version of the General Health Questionnaire (16), consisting of 12 questions (GHQ-12), taking into account the last two weeks. The questions concerned the quality of sleep, the feeling of being nervous or tense, the ability to maintain attention, the feeling of being useful, the ability to face problems, the ability to make decisions, the ability to overcome difficulties, the feeling of being happy, the satisfaction of accomplishing daily tasks, the feeling of being sad or depressed, the feeling of being self-confident, and having self-esteem. Each question had four options: **1)** *not at all*; **2)** *no more than usual*; **3)** *a little more than usual*; **4)** *much more than usual*. The scores of the individual items were coded as absent (*not at all* and *not more than usual*) or present (*a little more than usual* and *much more than usual*), and students who scored three or more positive answers were classified as having CMD (17).

### Work (Exposure)

The information related to work was collected through four questions: The first concerned paid work, the second concerned unpaid work, the third concerned the number of hours worked, and the last one concerned possible work accidents. All questions had an option in case the adolescent did not work. As the answers should take into account the experiences of the past year, the first and second questions (on paid and unpaid work) allowed the participant to mark more than one answer (company employee, household employee, intern, or freelancer). This study used data related to the form of work (paid or unpaid), number of hours worked, and possible work accidents.

### Covariates

The clinical and sociodemographic covariates considered in this study were gender, age, pubertal development on the Tanner scale (18), self-reported skin color (white, black, mulatto, Asian, indigenous), type of school (public or private), school year (7^th^ to 9^th^ grade of elementary school and 1^st^ to 3^rd^ year of high school), study shift (morning or afternoon), parents’ education (mother, father, and head of household), people with whom the adolescent resides (father, mother, both or none), and high blood pressure measured by the oscillometric method (systolic or diastolic blood pressure was above the 95^th^ percentile).

### Data Analysis

The data analysis considered the complex sampling structure of the ERICA study, as it used stratification and conglomeration in its selection stages, and was performed using Stata 14.0 statistical software (Survey module). The descriptive statistics consisted of the mean (standard deviation) for continuous variables and the absolute frequency (percentage) for categorical variables.

The associations between the variables included in the study with the primary outcome (CMD) were investigated by univariate and multivariate logistic regression models with an estimate of the odds ratio (OR) and their respective confidence intervals of 95% (95%CI). In all analyses, a significance level of 5% was considered, maintaining all variables with *p*<0.05 in the multivariate model.

## RESULTS

The main characteristics of the adolescents included in this study are shown in Table 1. The sample consisted of 3424 adolescents with a mean age of 14.2 years; it showed a balanced distribution between genders (50.72% male) and the participants were predominantly mixed-race (46.77%). Most participants studied in public schools (72.67%) and attended the morning shift (89.89%), with 61.71% of the participants in the last three years of elementary school. Most were at puberty in the stage of sexual maturation (Tanner 2, 3, and 4: 61.12%). Regarding the educational level of the parents or guardians, the most prevalent was tertiary education among fathers (36.76%) and secondary education among mothers (39.35%).

The prevalence of CMD among adolescents was 28.72%. A minority of adolescents reported working at the time of the interview (9.78%), and of these, only a small percentage (0.43%) worked above the legal workload for this age group (>30 hours). The proportion of those who reported performing paid or unpaid work in the last year totaled 19.63%, with a prevalence of work-related accidents or illnesses of 1.47% (Table 1). The percentage of CMD among adolescents who were in paid or unpaid work was 35.66%, and was higher in those who reported having had a work-related accident or illness in the last year (55.37%).

The associations between the variables included in the present study and the presence of CMD are shown in Tables 2 and 3. In the univariate analysis, the CMD was associated with age (OR 1.13; 95%CI 1.05-1.22), having an illiterate mother (OR 2.22; 95%CI 1.40-3.50), residing only with their mother (OR 1.39; 1.13-1.71), being female (OR 2.60; 2.01-3.37), at Tanner stages 4 or 5 of sexual maturation (OR 1.62; 1.18-2.22), having worked in the past year (OR 1.50; 1.18-1.90), and having had a work-related accident or illness (OR 3.19; 1.40-7.26). However, only female gender (OR 2.72; 95%CI 2.10-3.52) and total work (OR 1.70; 95%CI 1.32-2.18) remained associated with CMD in the multivariate analysis model.

## DISCUSSION

The main finding of the present study was an important independent association between CMD with work and female gender in school adolescents. Other variables that were associated with the presence of CMD among adolescents in the univariate analysis were age, mother’s education, residing only with mother, stage of sexual maturation, and the occurrence of any work accident in the last year, but these lost significance after adjustments for potential confounding variables.

The results of the present study are similar to those of a previous study conducted with 1211 participants aged over 15 years, which indicated a significant association between CMD, age, and female gender (19). The association between CMD and age may be justified by an increase in the prevalence of psychiatric disorders, chronic diseases, and exposure to stressful situations that are common with the advancement of age (20). Other researchers have also observed a higher prevalence of CMD among women, which may be associated with low education, poverty, domestic violence, and gender disadvantage (21,22). Previous studies have also found an association between female gender and CMD among school adolescents (23).

The association between low maternal education and CMD described in the present study has also been observed by other researchers (24). In this context, the shorter the schooling time, the lower the socioeconomic level, with the latter being a risk factor observed for the development or maintenance of CMD (25). In addition, not living with parents has also been described as a possible social characteristic associated with CMD (23). However, in the present study, we found an association only among those adolescents who did not live with their mother, reinforcing the importance of the mother in the context of the adolescent’s family (26).

The Tanner pubertal development stages 4 and 5, which represent adolescents in the final phase of sexual maturation and post-pubertal, respectively, were also associated with the presence of CMD. Since Tanner’s stages reflect human development, this association is probably related to the fact that the last two stages also represent older adolescents. In addition, the physical changes that occur during puberty may bring insecurity regarding acceptance from their peers, predisposing them to the occurrence of CMD (27).

In the present study, a significant association between CMD and work-related characteristics was observed, confirming the findings of other studies conducted in adult populations (28,29). This association can be explained by the high level of psychological demand present in some types of work, dissatisfaction with remuneration, lack of physical or leisure activities, and low social support (30-32). In addition, a history of work-related accidents was also associated with CMD, as has been shown in other studies in adult populations (33). A study with 354 adolescent workers of various functions found a high report of body aches, a high incidence of accidents, and reduction in sleep duration on weekdays among those who worked, concluding that work activities in this age group should be directed to learning and not to laboring as an adult (34). As for work-related diseases, some have a direct connection with the work environment, such as situations of high demand, hard or dangerous occupations, low professional achievement, and responsibility for the lives of others. Moreover, depending on the meaning that the work has in the life of the subject, it will generate psychological distress, which can explain the relationship between illnesses and work-related accidents with CMD in adolescents (35).

In the multivariate analysis, only female gender and work remained associated with CMD. In the adult population, the association between work and mental health in women can be explained by the fact that the work environment is usually affected by social and cultural factors, with different work experiences between genders and a pronounced disadvantage for women due to their higher workload, since women in Brazil are primarily responsible for domestic work and family care (36). As a sample of adolescents were evaluated in this study, perhaps this result indicates that gender inequality; in particular, the work overload on women, is already present even before the social and cultural roles of men and women are defined. In this setting, multi-component school-based interventions including innovative strategies (*e.g.* sports participation and positive development strategies) involving not only students but also school staff and parents may increase the psychosocial health, wellbeing, and quality of life of students and decrease school dropout rates (37,38).

The main limitation of this study is that the data for CMD were collected using an instrument that verifies various symptoms without necessarily pointing to a diagnosis. However, the use of this type of instrument was necessary because data collection was performed at a single moment, and therefore it would not be possible to apply a battery of tests. However, the results are consistent with those found in the literature.

To conclude, in the present study, a high number of cases of CMD were observed among the studied adolescents. Female gender and work history in the previous year were independently associated with the presence of CMD. Thus, new research that further assesses the various factors associated with the presence of CMD among adolescents in the job market is critical for developing strategies for its prevention, identification, and treatment in this group.

## AUTHOR CONTRIBUTIONS

All authors included in this manuscript provided significant contributions to the work. Santos DB, Mediano MFF, Santos Junior B and Kuschnir MCC contributed to the study conception, design, analysis and interpretation of data. Santos DB, Mediano MFF, Rodrigues Junior LF, Santos Junior B, Lorenzo AR and Kuschnir MCC revised the manuscript critically for important intellectual content, and Santos DB, Mediano MFF, Rodrigues-Junior LF, Santos Junior B, Lorenzo AR and Kuschnir MCC approved the final version if the manuscript.

## Figures and Tables

**Table 1:** Characteristics of adolescents included in the study (n=3424).

VARIABLES	FREQUENCY (%)
Sex	
Female	49.28
Male	50.72
Sexual maturation	
Tanner Stage 1	0.29
Tanner Stage 2	4.43
Tanner Stage 3	18.51
Tanner Stage 4	38.18
Tanner Stage 5	38.59
Skin color (n=3364)	
White	39.46
Black	11.22
Mixed-Race	46.77
Asian	2.07
Indigenous	0.47
School administration	
Public	72.67
Private	27.33
Educational level	
Primary school (7^th^, 8^th^, and 9^th^ grades)	61.71
Secondary school (1^st^, 2^nd^, and 3^rd^ grades)	38.29
School shift	
Morning	89.89
Afternoon	10.11
Father’s educational level (n=893)	
Illiterate	1.55
Primary	27.70
Secondary	33.99
Tertiary	36.76
Mother’s educational level (n=2500)	
Illiterate	1.65
Primary	26.40
Secondary	39.35
Tertiary	32.60
Householder’s educational level (n=2313)	
Illiterate	2.00
Primary	28.86
Secondary	33.66
Tertiary	35.48
Parents living with the adolescent	
Mother	36.22
Father	4.88
Both parents	54.06
No parents	4.84
Paid work in the last year	
No	84.92
Yes	15.08
Unpaid work in the last year	
No	88.22
Yes	11.78
Paid or unpaid work in the last year	
No	80.37
Yes	19.63
Current work hours per week (n=3353)	
No current work	90.22
≤30 hours	9.36
>30 hours	0.43
Work-related injury or illness in the last year (n=3386)	
No	21.51
Yes	1.47
Blood pressure	
Normal	92.68
High	7.32
Common mental disorder	
No	71.28
Yes	28.72

**Table 2:** Univariate associations between variables included in the study and common mental disorders.

VARIABLE	OR (95%CI)	*p*-value
Age (years)	1.13 (1.05 to 1.22)	0.001
Female sex	2.60 (2.01 to 3.37)	<0.001
Non-white	1.20 (0.95 to 1.51)	0.13
Sexual maturation (Tanner 4 and 5)	1.62 (1.18 to 2.22)	0.003
Private school administration	0.80 (0.63 to 1.03)	0.08
Illiterate mother	2.22 (1.40 to 3.50)	<0.001
Illiterate father	0.55 (0.13 to 2.34)	0.42
Illiterate householder	1.71 (0.69 to 4.30)	0.24
Living only with mother	1.39 (1.13 to 1.71)	0.02
Living only with father	0.91 (0.55 to 1.50)	0.71
Not living with parents (mother and father)	1.48 (0.87 to 2.52)	0.14
High blood pressure	0.87 (0.59 to 1.28)	0.48
Paid work in the last year	1.52 (1.15 to 1.99)	0.003
Unpaid work in the last year	1.55 (1.15 to 2.08)	0.004
Paid or unpaid work in the last year	1.50 (1.18 to 1.90)	0.001
Work-related injury or illness in the last year	3.19 (1.40 to 7.26)	0.006

**Table 3:** Multivariate association between sex and work (paid or unpaid) in the last year with common mental disorders.

VARIABLE	OR (95%CI)	*p-*value^1^
Female sex	2.72 (2.10 to 3.52)	<0.001
Paid or unpaid work in the last year	1.70 (1.32 to 2.18)	<0.001

1 Multivariate models were adjusted to those variables that were statistically significant in the univariate analysis (age, sex, sexual maturation (Tanner 4 and 5), illiterate mother, and living only with mother).
